# Reverted exhaustion phenotype of circulating lymphocytes as immune correlate of anti-PD1 first-line treatment in Hodgkin lymphoma

**DOI:** 10.1038/s41375-021-01421-z

**Published:** 2021-09-28

**Authors:** Maria A. Garcia-Marquez, Martin Thelen, Sarah Reinke, Diandra Keller, Kerstin Wennhold, Jonas Lehmann, Johanna Veldman, Sven Borchmann, Andreas Rosenwald, Stephanie Sasse, Arjan Diepstra, Peter Borchmann, Andreas Engert, Wolfram Klapper, Michael von Bergwelt-Baildon, Paul J. Bröckelmann, Hans A. Schlößer

**Affiliations:** 1 Faculty of Medicine and University Hospital of Cologne, Center for Molecular Medicine Cologne (CMMC), University of Cologne, Cologne, Germany; 2 Institute of Pathology, University of Kiel, Kiel, Germany; 3 Department of Pathology and Medical Biology, University of Groningen, University Medical Center Groningen, Groningen, The Netherlands; 4 Faculty of Medicine and University Hospital of Cologne, Department I of Internal Medicine, Center for Integrated Oncology Aachen Bonn Cologne Düsseldorf (CIO ABCD), German Hodgkin Study Group (GHSG), University of Cologne, Cologne, Germany; 5 Department of Pathology, University of Würzburg, Würzburg, Germany; 6 Department of Internal Medicine III, University Hospital, Ludwig Maximilians University, Munich, Germany; 7 Mildred Scheel School of Oncology Aachen Bonn Cologne Düsseldorf (MSSO ABCD), Faculty of Medicine and University of Cologne, Cologne, Germany; 8 Max Planck Institute for Biology of Ageing, Cologne, Germany; 9 Faculty of Medicine and University Hospital Cologne, Department of General, Visceral, Cancer and Transplantation Surgery, Center for Integrated Oncology Aachen Bonn Cologne Düsseldorf (CIO ABCD) and University of Cologne, Cologne, Germany

**Keywords:** Hodgkin lymphoma, Immunotherapy, Lymphocytes, Immunosurveillance

## Abstract

While classical Hodgkin lymphoma (HL) is highly susceptible to anti-programmed death protein 1 (PD1) antibodies, the exact modes of action remain controversial. To elucidate the circulating lymphocyte phenotype and systemic effects during anti-PD1 1st-line HL treatment we applied multicolor flow cytometry, FluoroSpot and NanoString to sequential samples of 81 HL patients from the NIVAHL trial (NCT03004833) compared to healthy controls. HL patients showed a decreased CD4 T-cell fraction, a higher percentage of effector-memory T cells and higher expression of activation markers at baseline. Strikingly, and in contrast to solid cancers, expression for 10 out of 16 analyzed co-inhibitory molecules on T cells (e.g., PD1, LAG3, Tim3) was higher in HL. Overall, we observed a sustained decrease of the exhausted T-cell phenotype during anti-PD1 treatment. FluoroSpot of 42.3% of patients revealed T-cell responses against ≥1 of five analyzed tumor-associated antigens. Importantly, these responses were more frequently observed in samples from patients with early excellent response to anti-PD1 therapy. In summary, an initially exhausted lymphocyte phenotype rapidly reverted during anti-PD1 1st-line treatment. The frequently observed IFN-y responses against shared tumor-associated antigens indicate T-cell-mediated cytotoxicity and could represent an important resource for immune monitoring and cellular therapy of HL.

## Introduction

Classical Hodgkin Lymphoma (HL) is a B-cell-derived hematologic malignancy characterized by a unique tumor microenvironment (TME). The HL TME is composed mainly of noncancerous immune cells, such as T cells, B cells, eosinophils, and macrophages that are recruited by the scarce malignant Hodgkin and Reed-Sternberg (HRS) cells [1–3]. Besides the characteristic CD30 positivity, HRS cells frequently express the co-inhibitory molecule programmed death ligand 1 (PD-L1).

Drugs targeting the PD1/PD-L1 axis such as the anti-PD1 antibodies nivolumab and pembrolizumab have demonstrated outstanding efficacy in relapsed/refractory (r/r) HL, and are approved in this setting [4–6]. While primary and secondary resistance is a major challenge in most solid cancers [7–9], the vast majority of HL patients responds to anti-PD1 therapy and durable remissions are observed. Based on the currently available data, HL is probably the malignant disease with the highest sensitivity to immune-checkpoint inhibition [10, 11]. Recently, the German Hodgkin Study Group (GHSG) phase II NIVAHL trial showed remarkable efficacy with either concomitant or sequential first-line nivolumab and doxorubicin, vinblastine, and dacarbazine (AVD) chemotherapy in early-stage unfavorable HL [12, 13]. Similarly striking efficacy was also observed with sequential pembrolizumab and AVD in patients with early-stage unfavorable or advanced-stage disease [14] and with concomitant nivolumab and AVD in advanced-stage HL [15]. However, despite the effectiveness of anti-PD1 therapy seen in HL, the mechanism of action remains controversial.

The stage-dependent high PD-L1 expression observed in HL, either due to genetic amplification of the 9p24.1 locus or EBV infection, partially correlates with sensitivity to anti-PD1 treatment [16–18]. Nevertheless, the high responsiveness is surprising as other aspects of HL biology are not in favor of increased anti-PD1 sensitivity. Most importantly, 40–60% of HL cases contain mutations of beta-2-microglobulin (ß2M) leading to impaired or complete loss of HLA-I expression [19, 20]. This is contradictory to the observed very good response rates, as impaired HLA-I-restricted peptide presentation leads to reduced or abolished immunorecognition by cytotoxic T cells, a crucial mechanism of anti-PD1 therapy, at least in solid tumors [21, 22]. Accordingly, a recent study of sequential biopsies taken only days after initiation of nivolumab-based first-line treatment did not find evidence of a CD8 T-cell mediated cytotoxic immune response despite very early histologic complete response [23]. In addition, anti-PD1 therapy predominantly affected the CD4 T-cell compartment in HL in a recent publication using cytometry by time of flight (CyTOF) analyses of longitudinal samples from patients who received nivolumab for r/r HL [24].

Data from clinical trials using immune-checkpoint inhibition in melanoma and lung cancer showed an association of specific changes in circulating lymphocyte subsets to clinical outcome [25–27]. Upregulation of co-inhibitory molecules in the TME of HL has been described as correlate of T-cell exhaustion [1], but the impact of HL on the circulating lymphocyte compartment and its changes during anti-PD1 first-line therapy are poorly described.

With samples obtained within the translational research program of the GHSG NIVAHL trial, we herein aim to comprehensively characterize the peripheral immune signature with a focus on lymphocyte subsets and immune-checkpoint molecules in HL. Our study is the first to report a detailed flow-cytometric assessment of baseline parameters and the evolution of circulating lymphocyte subsets as well as tumor-specific immune responses in a large well-defined HL cohort during innovative anti-PD1 first-line treatment.

## Materials and methods

### Patients and samples

Peripheral blood samples of 81 patients undergoing experimental nivolumab-based first-line treatment for early-stage unfavorable HL were analyzed as part of the translational research program of the prospective randomized multicenter GHSG NIVAHL phase II trial (NCT03004833). Patient characteristics, study treatment, and outcomes of the whole study have been reported elsewhere [12, 28]. Briefly, HL patients either received 4 cycles of fully concomitant nivolumab plus AVD chemotherapy (4× Nivo-AVD) or sequential treatment with 4× nivolumab, 2× Nivo-AVD and 2× AVD, each followed by 30 Gy involved-field radiotherapy (IF-RT). PBMCs were collected before treatment (BT) (timepoint 0, TP0), after the first nivolumab dose (TP1), at first restaging after 4× nivolumab (TP2), and the second restaging after the end of systemic therapy prior to IF-RT (TP3). PBMCs were isolated by density centrifugation using PANCOLL (PAN-Biotech, Germany), resuspended in FBS + 10% DMSO and stored in liquid nitrogen until analysis. Written informed consent for the additional translational program was obtained from all patients included in the analyses and patient and sample availability are summarized in Supplementary Fig 1 (Consort Diagram). Healthy controls also provided written informed consent and this study was approved by the Ethics Committee of the University of Cologne (patients: no. 16-444, healthy controls: no. 11-116).

### Flow cytometry

PBMCs were stained for multicolor flow cytometry for 20 min at 4 °C using a comprehensive panel of antibodies to identify main immune cell subsets and potentially actionable co-regulatory molecules (Supplementary Table 1). Dead cells were excluded using a viability dye (Biolegend, USA). Foxp3 staining was performed using the Foxp3/Transcription Factor Staining Buffer Set (Invitrogen, USA) according to the manufactures' protocol. Data were acquired on a Cytoflex LX flow cytometer (Beckman Coulter, USA) and analyzed using the Kaluza software v.2.1 (Beckman Coulter, USA).

### FluoroSpot assay

A total of 2 × 10^5^ PBMC were incubated in pre-coated 96-well plates (Mabtech, Sweden) with optimized peptide pools of the tumor-associated antigens (TAAs) selected based on previous publications and publicly available databases (proteinatlas.org) describing their expression in HL [2, 3, 29–32]. Five peptide pools (whole protein, 15mers with 11 aa overlap) were selected BMLF-1, PRAME, MAGE-A4, MAGE-C1, and EBNA-1 (peptide&elephants, Germany), and were used in a concentration of 1 µg/ml per peptide. As control peptide, an optimized peptide pool from sequences derived from the human Cytomegalovirus (CMV, peptide & elephants, Germany) was applied. An anti-CD3 antibody was used as positive control at a dilution of 1:1000. All stimuli contained an anti-CD28 antibody in a dilution of 1:1000 for co-stimulation of T cells. PBMCs in serum-free AIM-V medium (ThermoFisherScientific, USA) were incubated with the stimuli at 37 °C for 20 h. After incubation, released cytokines were detected with specific antibodies and fluorescent secondary antibodies contained in the kit (Mabtech, Sweden). Plates were read with an AID EliSpot reader (Autoimmun Diagnostika, Germany) and analyzed with the EliReader Software. FluoroSpot assays were performed in triplicates.

### Immunohistochemistry

Baseline HL biopsies were stained for ß2M (main mechanism of HLA-I loss in HL) and HLA-II (HLA-DP/DQ/DR) and LMP1 (EBV status; Supplementary Table 1) according to standard IHC protocols. ß2M and HLA-II IHC were scored independently by two experienced hematopathologists (WK and AD), and discrepancies were consented in a joint review. Cases were assessed for membranous ß2M and HLA-II staining according to categories previously published [33] and categorized as positive (>50% of the tumor cells, HRSC+) or negative (heterogeneous staining of the tumor cells (HRSC±) and membranous staining in < 50% of the tumor cells; HRSC−).

### RNA isolation and NanoString

RNA-extraction and gene expression profiling were performed on all primary tumor biopsies as previously described using the NanoString (nCounter, Nanostring, USA) technology and the PanCancer Immune Panel (nCounter, NanoString, USA) [34]. NanoString data were available for all patients included in this analysis processed and analyzed using the nSolver software (Nanostring, USA) according to the manufacturer’s instructions as previously described [34].

### Statistical analyses and visualizations

Data were analyzed using GraphPad Prism v.9.0.2 (GraphPad, USA). Figures were generated using Inkscape v1.0.1. Group sizes, levels of statistical significance, definition of error bars, and applied tests are included in figure legends. Similarity matrices and hierarchical clustering were generated using MORPHEUS (Morpheus by Broad Institute, USA).

### Data sharing statement

The data generated and analyzed during the present study can be made available upon reasonable request to the corresponding authors.

## Results

### Circulating T cells show an exhausted phenotype in baseline samples of HL patients

Disease-specific changes in peripheral blood of patients with early-stage unfavorable HL were assessed by flow cytometry. We compared the composition of lymphocyte subsets and expression of immune-regulatory molecules in peripheral blood of 72 treatment-naive baseline samples to results from peripheral blood of 20 healthy controls (Gating strategy in Supplementary Fig. 2; the mean age and range of HL patients and healthy controls was 31 years (18–57) and 54 years (26–65), respectively.).

**Fig. 1:**
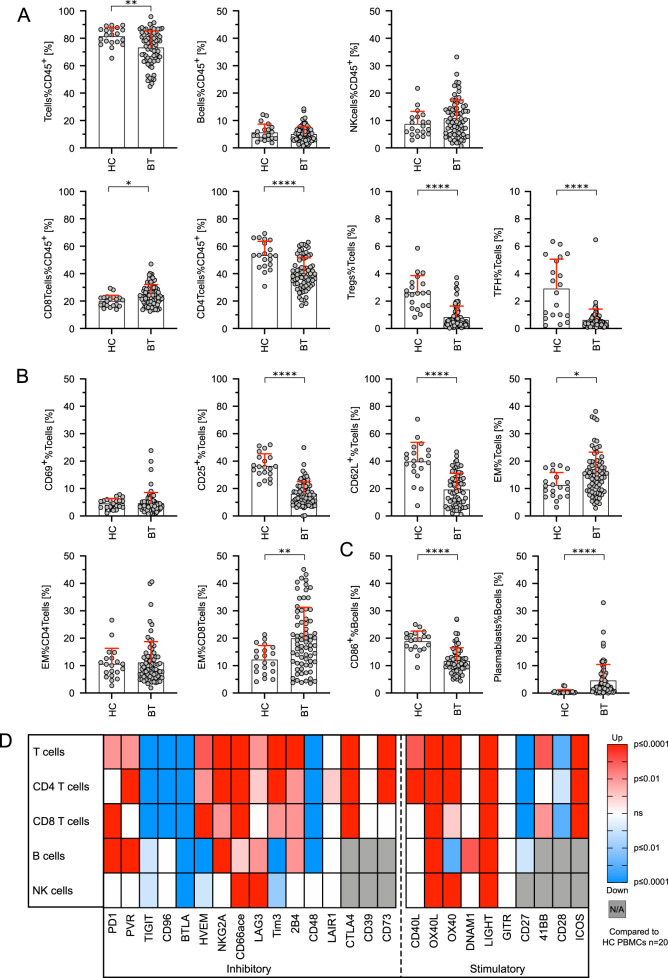
Flow-cytometric analyses revealed specific changes in lymphocyte subsets and increased expression of immune-regulatory molecules on B, T and NK cells in PBMCs of treatment-naive HL patients. **A** Lymphocyte subsets in peripheral blood mononuclear cells (PBMCs) of Hodgkin lymphoma (HL) patients before therapy (BT, *n* = 72) and healthy donor PBMCs (HC, *n* = 20) were analyzed by flow cytometry. **B** Differences in T-cell activation (CD69^+^%Tcells, CD25^+^%Tcells, and CD62L^+^%Tcells) and T-cell differentiation (CCR7^-^CD45RA^-^ effector memory (EM) T cells) of HL patients before therapy (BT) and healthy control PBMCs (HC). **C** Differences in B-cell activation (CD86^+^%Bcells) and B-cell differentiation (plasmablasts%Bcells) of HL patients before therapy (BT) and healthy control PBMCs (HC). **D** Immune-regulatory molecule expression was determined by flow cytometry. Significant up/downregulation of co-inhibitory and co-stimulatory molecule expression on T cells, CD4 T cells, CD8 T cells, B cells, and NK cells of HL patients before therapy (*n* = 72) compared to the mean expression obtained from healthy control PBMCs (*n* = 20). Significant differences calculated by unpaired, two-tailed Mann–Whitney test are indicated by asterisks. *p ≤ 0.05, **p ≤ 0.01, ***p ≤ 0.001, ****p ≤ 0.0001. When appropriate, mean ± SD is indicated.

We found reduced fractions of T cells (Tcells%CD45^+^) in HL (73.3% ± 12.1 vs. 81.4% ± 6.4, *p* < 0.01), mainly CD4^+^ T cells (40.2% ± 11.5 vs. 53.5% ± 10.1 of CD45^+^ lymphocytes, *p* < 0.01). CD3^+^CD4^+^CD25^++^FoxP3^+^ regulatory T cells (0.8% ± 0.8 vs. 2.6% ± 1.2 of CD3^+^ T cells, *p* < 0.01) and CD4^+^CD45RA^−^CXCR5^+^ T follicular helper cells as major CD4^+^ T cells subsets were also significantly lower in HL patients (0.6% ± 0.8 vs. 2.9% ± 2.1, *p* < 0.01). We did not observe significant differences regarding the relative fraction of CD8^+^ cytotoxic T cells (CD8Tcells%CD45^+^), B cells (Bcells%CD45^+^), and natural killer cells (NKcells%CD45^+^) (Fig. 1A). Fractions of T cells expressing the early activation marker CD69 were similar, whereas T cells expressing the late activation markers CD25 (16.2% ± 8.8 vs. 36.6% ± 8.8, *p* < 0.01) and CD62L (19.3% ± 11.9 vs. 39.8% ± 14.0, *p* < 0.01) were lower in patients with HL compared to healthy controls (Fig. 1B). The percentage of CD45RA^−^CCR7^−^ effector-memory (EM) T cells however was higher in PBMCs of HL patients (15.5% ± 7.8 vs. 11.3% ± 4.6, p < 0.05). While the CD4^+^ fractions of EM T cells were similar, we found a significant increase of CD8^+^ EM T cells in patients with HL (19.9% ± 11.4 vs 12.2% ± 5.2, *p* < 0.01) (Fig. 1B). Accordingly, the naive CD4^+^ T-cell subset did not differ significantly from healthy controls (*p* = 0.0946) and we observed significantly fewer naive CD8^+^ T cells in HL patients (*p* < 0.05; Supplementary Fig. 3A). The percentage of B cells (CD19^+^CD20^+^ lymphocytes) expressing CD86 as marker of activation was also reduced in HL compared to healthy controls (11.7% ± 4.8 vs. 18.9% ± 3.7, *p* < 0.01) while we found an increase of CD20^-^CD38^++^ plasmablasts (4.6% ± 5.8 vs. 0.5% ± 0.8, *p* < 0.01) (Fig. 1C). We observed a trend towards fewer NK cells in HL patients and found a significantly reduced CD56^bright^ population (Supplementary Fig. 3B). We did not find any relevant differences in the peripheral immune signature of EBV^+^ vs. EBV^−^ cases (Supplementary Fig. 4).

**Fig. 2:**
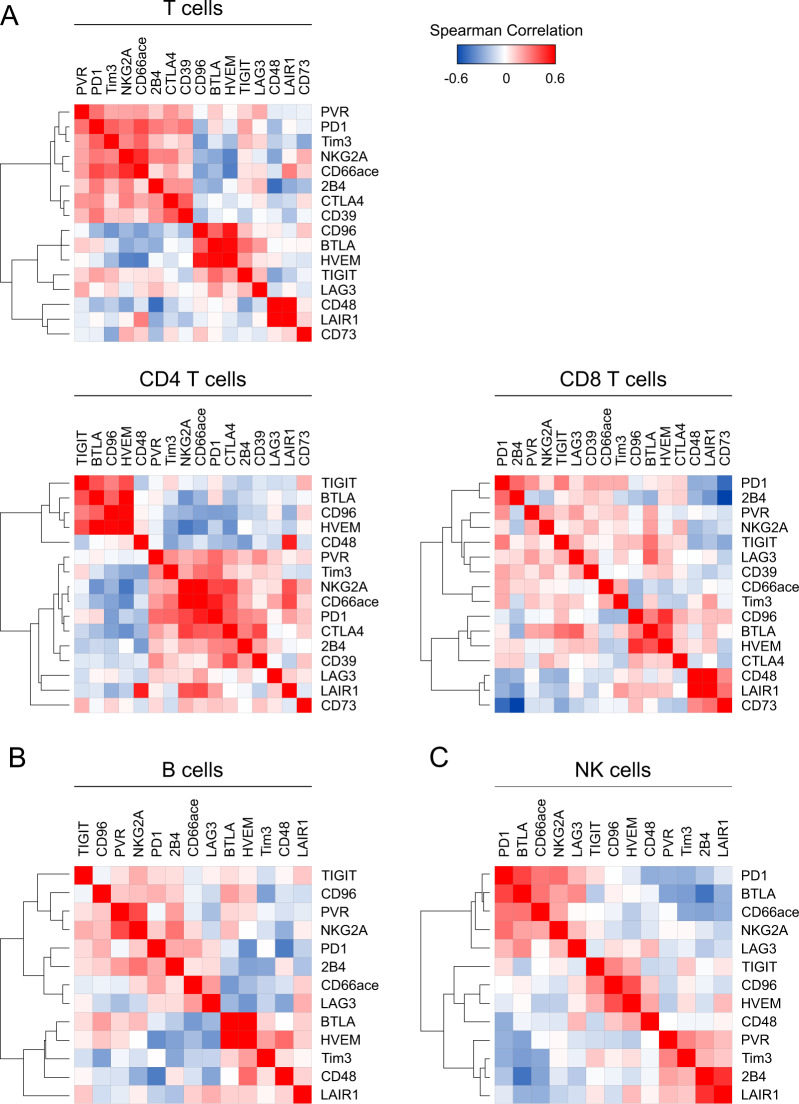
Immune-inhibitory molecules are co-expressed on T cells but also on B and NK cells. Expression of the indicated immune-inhibitory molecules on peripheral blood mononuclear cells (PBMCs) of Hodgkin lymphoma (HL) patients before therapy was determined using flow cytometry. Similarity matrices are showing correlations of immune-inhibitory molecules on T cells, CD4 T cells, CD8 T cells (**A**), B cells (**B**), and NK cells (**C**). Heatmap was generated by spearman rank correlation followed by one minus spearman rank correlation using average linkage for hierarchical clustering.

Assessment of 26 co-inhibitory and co-stimulatory molecules was included into our flow-cytometric analyses to assess immune cell activation and exhaustion states. These molecules have previously been studied extensively and are frequently associated with an exhausted phenotype due to limited cytotoxicity and reduced re-expansion capacity [9, 35–38]. In T cells, we found increased expression of 10/16 co-inhibitory molecules (62.5%), while 2/16 molecules (12.5%; LAIR1 and CD39) were unchanged and 4/16 (25.0%; TIGIT, CD96, BTLA, and CD48) reduced in HL patients compared to healthy controls (Fig. 1D). The increased fraction of cells expressing the co-inhibitory molecules PVR, LAG3, and CD73 observed in the whole T-cell subset was not found for CD8^+^ T cells. Differences regarding expression of co-inhibitory molecules were less pronounced on B and NK cells. For B cells, subsets expressing 6/13 (46.1%) immune-regulatory molecules were increased, 2/13 (15.3%) were unchanged and 5/13 (38.4%) were lower in samples from HL patients. Fractions of NK cells expressing 2/13 (15.3%) analyzed co-inhibitory molecules were higher, 7/13 (53.8%) were unchanged and 4/13 (30.7%) were lower in HL patients compared to healthy controls. In addition, various co-stimulatory molecules were overexpressed in T, B, and NK cells prior to treatment compared to healthy controls (Fig. 1D). By hierarchical clustering of BT data, we found co-regulation of multiple molecules mainly on T cells (Fig. 2A) but also to a lesser extent on B and NK cells (Fig. 2B, C).

### Expression of immune-inhibitory molecules related to the observed exhausted phenotype rapidly decreased in HL patients treated with nivolumab

We aimed to elucidate anti-PD1 treatment-induced changes in the composition of circulating lymphocyte subsets, their activation, differentiation, and expression levels of immune-regulatory molecules. We therefore additionally analyzed sequential samples obtained 1 (timepoint 1, TP1) and 8 (TP2) weeks after treatment (AT) initiation, respectively, and after completion of systemic treatment with in total 8× nivolumab and four AVD cycles in both groups (TP3).

**Fig. 3:**
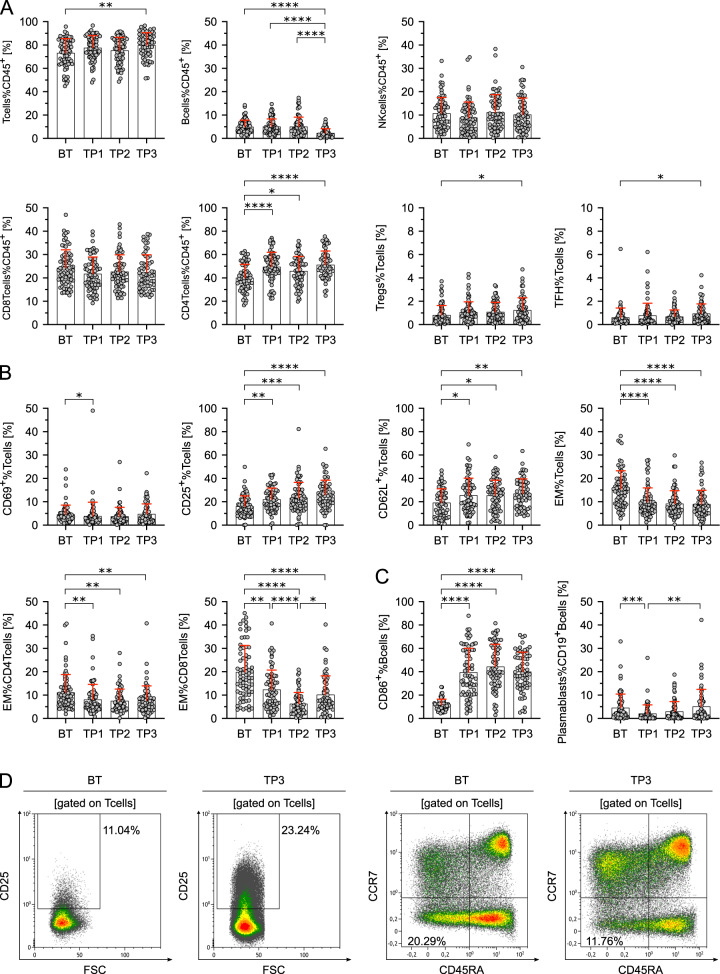
The observed effects of HL on composition and maturation of peripheral lymphocytes are reversed following anti-PD1 therapy. **A** Lymphocyte subsets in percent of CD45^+^ lymphocytes or T cells in peripheral blood mononuclear cells of Hodgkin lymphoma (HL) patients before therapy (BT, *n* = 72), sequential samples obtained 1 and 8 weeks after treatment initiation (TP1, *n* = 72 and TP2, *n* = 68) and after completion of systemic treatment (TP3, *n* = 62). Samples containing ≤100 CD45^+^ cells were excluded. **B** Differences in T-cell activation (CD69^+^%Tcells, CD25^+^%Tcells, and CD62L^+^%Tcells) and T-cell differentiation (CCR7^−^CD45RA^−^ effector memory (EM) T cells) of HL patients before (BT) and during the course of therapy (TP1-TP3). **C** Differences in B-cell activation (CD86^+^%Bcells) and B-cell differentiation (plasmablasts%Bcells) of HL patients before therapy (BT) and during the course of therapy (TP1–TP3). **D** Representative flow cytometry plots of peripheral blood mononuclear cells obtained from a HL patient before therapy (BT) and after completion of treatment (TP3). CD25^+^ activated T cells (left two plots) and CCR7^−^CD45RA^−^ effector-memory T cells are shown (right two plots). Significant differences calculated by nonparametric Kruskal–Wallis test followed by Dunn’s post hoc test (**A**–**C**) are indicated by asterisks. *p ≤ 0.05, **p ≤ 0.01, ***p ≤ 0.001, ****p ≤ 0.0001. When appropriate, mean ± SD is indicated.

Whereas the relative fraction of NK cells remained unchanged, CD3^+^ T cells were higher and B cells lower in TP3 compared to BT samples. This was mainly due to increased CD4^+^ T cells already at TP1. Analyses of Tregs and TFH revealed increased percentages on TP3 (Fig. 3A). On T cells, the early activation marker CD69 decreased at TP1, and expressions of the activation markers CD25 and CD62L increased compared to baseline samples at all three analyzed timepoints (Fig. 3B, D). This was accompanied by a decrease of EM T cells overall, CD4^+^ and CD8^+^ EM T cells as well as CD45RA^−^CCR7^−^ EM T cells compared to BT samples (Fig. 3B, D). CD86 as key activation marker of B cells was increased compared to BT samples at all analyzed timepoints while percentages of plasmablasts remained largely unchanged (Fig. 3C). In addition to the increased expression of activation markers on T and B cells, we found a striking downregulation of co-inhibitory molecules upon treatment with nivolumab.

**Fig. 4:**
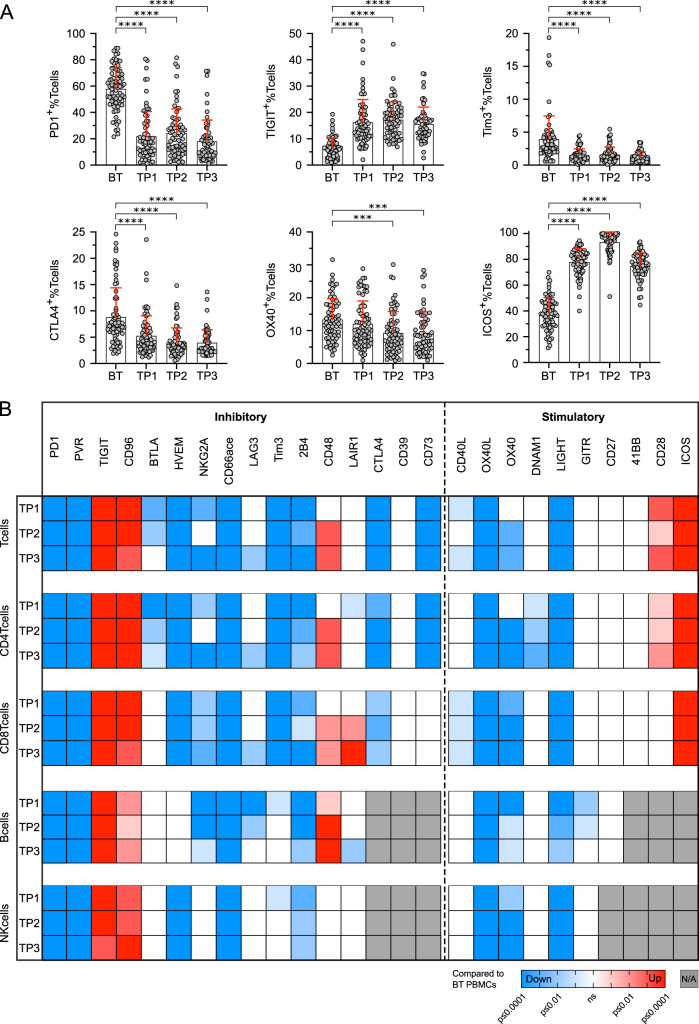
Anti-PD1 therapy reduces the expression of immune-regulatory molecules on PBMCs of HL patients. **A** Expression of immune-regulatory molecules was determined by flow cytometry analysis of peripheral blood mononuclear cells (PBMCs) isolated from Hodgkin lymphoma (HL) patients before therapy (BT, *n* = 72), sequential samples obtained 1 and 8 weeks after treatment initiation (TP1, *n* = 72 and TP2, *n* = 68) and after completion of treatment (TP3, *n* = 62). Samples containing ≤100 CD45^+^ cells were excluded. Selected plots showing PD1, TIGIT, Tim3, CTLA4, OX40, and ICOS expression on T cells of HL patients before therapy (BT) and during the course of therapy are depicted. **B** Significant up/ downregulation of co-inhibitory and co-stimulatory molecule expression on T cells, CD4 T cells, CD8 T cells, B cells, and NK cells of HL patients during therapy (T1–TP3) compared to the mean expression obtained from before therapy samples (*n* = 72). Significant differences of before therapy (BT) samples and samples during the course of therapy (TP1–TP3) were calculated by nonparametric Kruskal–Wallis test followed by Dunn’s post hoc test. Results are indicated by asterisks. *p ≤ 0.05, **p ≤ 0.01, ***p ≤ 0.001, ****p ≤ 0.0001. When appropriate, mean ± SD is indicated.

Expression of 10/16 (62.5%) analyzed co-inhibitory molecules showed a significant decrease on T cells for at least one timepoint after anti-PD1 treatment initiation (Fig. 4A, B). Only 2/16 (12.5%, TIGIT, and CD96), which were initially decreased compared to healthy controls, showed an increased expression in post-treatment samples. With few exceptions, these changes in expression of co-inhibitory molecules were similar for CD4^+^ and CD8^+^ T cells, both showing a rapid reversal of the exhausted phenotype observed in BT samples. Expression of co-stimulatory molecules showed fewer differences upon anti-PD1 treatment. Despite an increased expression of ICOS and CD28 (Fig. 4A), we did not observe further upregulation of co-stimulatory molecules on T cells compared to HC after start of treatment. 4/10 (40.0%) co-stimulatory molecules showed a decreased expression on T cells in at least one TP and we did not find significant differences for the remaining 4 co-stimulatory molecules (Fig. 4B). We also included expression of immune-regulatory molecules on B and NK cells into our analysis. Although less frequent than for T cells, we found a downregulation of several immune-regulatory molecules in post-treatment samples for these lymphocyte subsets as well (Fig. 4B).

### HL patients frequently show tumor-specific immune responses to shared tumor-associated antigens

In addition to changes in the described comprehensive phenotyping, we were interested in tumor-specific immune responses of circulating lymphocytes in HL. Analyses of T-cell responses to the shared TAAs EBNA-1, BMLF-1, MAGE-A4, MAGE-C1, and PRAME in FluoroSpot assays was feasible in a total of 71 NIVAHL patients with sufficient cell number.

**Fig. 5:**
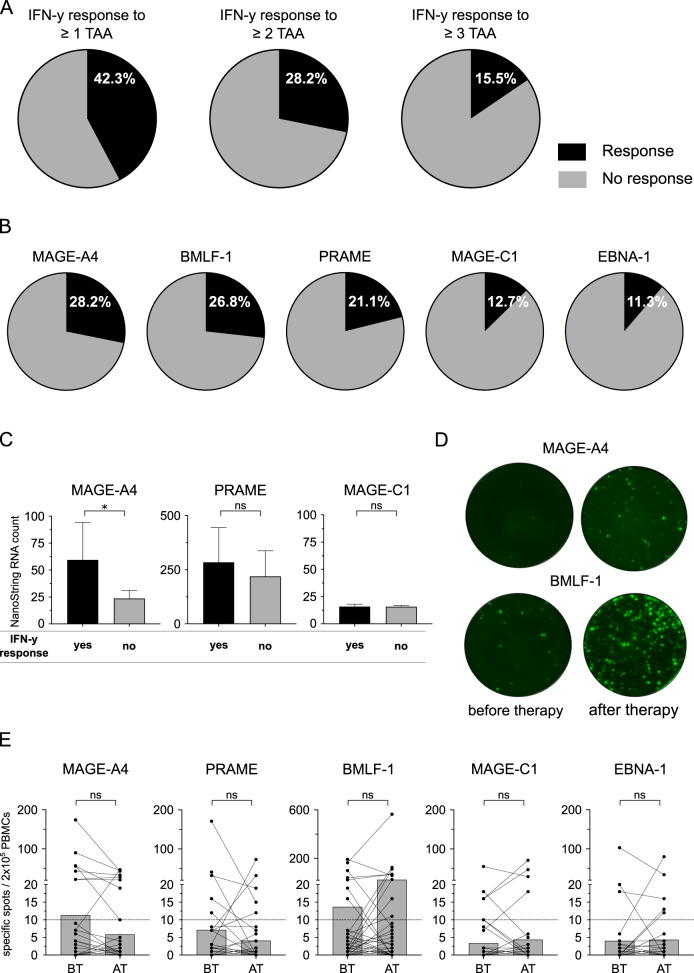
HL patients frequently show immune responses against tumor-associated antigens. **A** 2 × 10^5^ peripheral blood mononuclear cells (PBMCs) of Hodgkin lymphoma (HL) patients before therapy or under therapy (*n* = 71) were co-cultured with peptide pools of five tumor-associated antigens (TAAs) separately. Interferon-gamma (IFN-y) secretion was detected by FluoroSpot assay. Percentage of HL patients showing an immune response (black) against ≥1 (left chart), ≥2 (center chart) or ≥3 (right chart) of the five tested TAAs (BMLF-1, PRAME, MAGE-A4, MAGE-C1, and EBNA-1) is depicted. **B** Percentages of patients showing an IFN-y response against the indicated TAAs. **C** RNA expression of TAAs was determined by NanoString. MAGE-A4, PRAME, MAGE-C1 expression in HL patients with (black) and without (gray) an IFN-y response (before or under therapy, *n* = 71) in FluoroSpot analyses. **D** Representative FluoroSpot plots showing IFN-y secretion after co-culture with the indicated TAA. IFN-y secretion for two HL patients in matched samples before and after therapy is shown. **E** FluoroSpot response in patient-matched PBMCs obtained from HL patients before (BT) and after therapy (AT). Number of specific spots in 2 × 10^5^ PBMCs after co-culture with the indicated peptide pools is depicted. Significant differences calculated by unpaired, two-tailed Mann–Whitney test (**C**) or nonparametric, two-tailed, Wilcoxon matched-pairs signed rank test (**E**) are indicated by asterisks. *p ≤ 0.05. When appropriate, mean ± 95% confidence interval is indicated.

While stimulation of PBMCs with TAA peptide pools only scarcely induced IL-10 and IL-5 specific spots, we found interferon-gamma (IFN-y) responses to at least one shared antigen in 30/71 patients (42.3%). Responses to ≥2 and ≥3 of the included TAAs were present in 20/71 (28.2%) and 11/71 (15.5%) patients, respectively (Fig. 5A). No significant differences were observed between the two treatment groups (Supplementary Fig. 5A–C). Tumor-specific immune responses were found for all included TAAs and most frequently observed for MAGE-A4 (28.2%; Fig. 5B). Comparison of gene expression levels of MAGE-A4, MAGE-C1, and PRAME revealed a significantly higher expression of MAGE-A4 in patients with MAGE-A4 specific immune response in FluoroSpot assays (Fig. 5C). The mean expression of PRAME was also higher in patients with IFN-y response, but differences were not significant for PRAME and MAGE-C1, which showed low expression in all patients (Fig. 5C).

Pre- and post-treatment blood samples of 45 patients contained sufficient viable cells to allow paired comparison of tumor-specific immune responses BT and AT with nivolumab (Supplementary Fig. 1). Our analyses revealed 14 de novo responses (not detectable BT; e.g. MAGE-A4 in Fig. 5D) and 18 persisting responses to the different shared antigens (e.g., BMLF-1 in Fig. 5D). However, we also observed ten losses of specific responses which were detectable in BT samples but not in subsequent AT samples (Fig. 5E). 14/45 (31.1%) and 13/45 (28.9%) patients showed IFN-y responses to at least one shared antigen in BT and AT samples, respectively (*p* > 0.99). The percentage of positive responses to the tested shared antigens was 11.4% (29/255) in BT and 12.2% (31/255) in AT samples (*p* = 0.68) and the mean number of specific spots also did not show significant differences (39.1 vs. 43.5, *p* = 0.88). In summary, we did not observe a significant increase regarding the number of specific responses to shared antigens in AT and BT samples.

### Tumor-specific immune responses to TAAs are more frequent in patients with early excellent response to nivolumab and not associated to ß2M/HLA-II expression

All but one NIVAHL patient remain in ongoing complete remission with the currently available follow-up [28]. Stratification of immunological data according to clinical outcome variables like PFS is hence not feasible. As also described in another recent phase II HL trial [14], an excellent early response with reduction of the initial metabolic tumor volume (MTV) by >90% was achieved at first restaging after 2× N-AVD (arm A) or 4× nivolumab (arm B) in many but not all patients [12, 13].

**Fig. 6:**
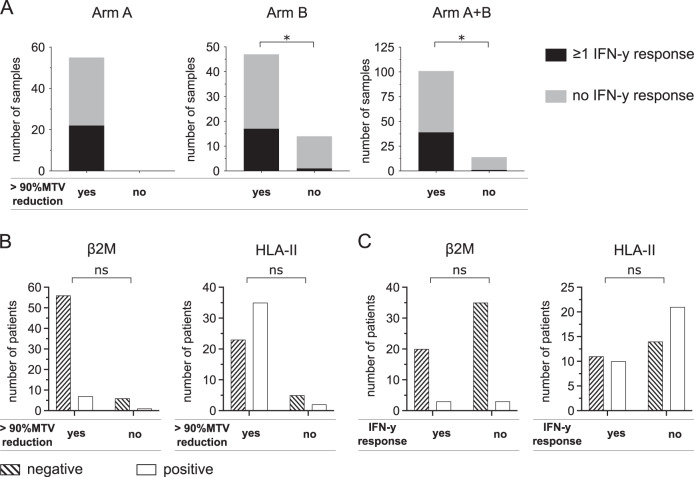
Association of early excellent response to anti-PD1 first-line HL treatment with IFN-y response to TAAs and HLA status. **A** 2 × 10^5^ peripheral blood mononuclear cells (PBMCs) of Hodgkin lymphoma patient (HL) before or under therapy were co-cultured with peptide pools of five tumor-associated antigens (TAAs) separately (BMLF-1, PRAME, MAGE-A4, MAGE-C1, and EBNA-1). Number of samples from patients of treatment arm A (left), arm B (center) and arm A + B (right) divided by >90%MTV reduction is indicated. Patients showing an interferon-gamma (IFN-y) response against at least one of the five tested antigens (black) or no IFN-y response (gray) in FluoroSpot assays are indicated. **B** beta-2 microglobulin (β2M)/HLA-II expression was assessed by immunohistochemistry. Number of β2M / HLA-II negative or β2 /HLA-II positive HL patients divided by 90% MTV reduction. **C** Number of β2M / HLA-II negative or β2M /HLA-II positive HL patients divided by IFN-y response in FluoroSpot assays. Significant differences calculated with two-sided Fisher’s exact test are indicated by asterisks. *p ≤ 0.05.

We stratified data of the HL patients according to >90% MTV reduction at first restaging after either 2× Nivo-AVD (arm A) or 4× nivolumab (arm B) to assess immune correlates of these excellent early responses. Patients with >90% MTV reduction had significantly more samples with IFN-y response to at least one TAA than patients achieving ≤90% MTV reduction, both in treatment arm B and the overall study (Fig. 6A). The fraction of patients with IFN-y response to at least one TAA was 45.7% (arm A), 48.2% (Arm B), and 46.8% (Arm A + B) in patients compared to 12.5% (Arm B and Arm A + B) in patients without >90% reduction in MTV (Supplementary Fig. 5D). Correlation of ß2M and HLA-II status with early excellent response to nivolumab-based 1st-line treatment did not show significant differences (Fig. 6B). Similarly, we did not detect any significant associations between ß2M/HLA status and IFN-y response to shared TAA (Fig. 6C) or any of the analyzed immune cell subsets or expression of co-regulatory molecules at baseline (Supplementary Table 2).

A recent study identified several tumor and T-cell intrinsic factors associated with sensitivity to immune-checkpoint inhibition in various solid tumors [39]. To elucidate the presence and prognostic role of these factors and TAAs in HL we conducted NanoString analysis on primary tumor biopsies and found high expression of CT45A1, PRAME, Survivin, and other TAAs (Supplementary Fig. 6). We observed a significantly increased expression of TAP1 in patients with >90% MTV reduction and increased IFN-y responses (Supplementary Fig. 7A). We additionally found a significantly increased expression of CCR5, CXCL9, and PD-L1 in patients with excellent early response to anti-PD1 first-line treatment (Supplementary Fig. 7B), while CD8A, CXCL13, and TRAF2 expression were not significantly associated with early efficacy.

## Discussion

Immunotherapy targeting the PD1 pathway demonstrated outstanding efficacy in HL. Our study represents one of the first prospective analyses of systemic effects of 1st-line anti-PD1 therapy in this disease. Comparison of lineages and phenotypes of lymphocytes in PBMC from baseline blood samples of HL patients and healthy controls revealed several differences. We found a reduction of the CD3^+^ T-cell subset, which was mainly due to decreased CD4^+^ T cells. The previously described intratumoral accumulation of “rosetting” CD4 T cells [40], hence appears to be accompanied by a reduction of circulating T helper cells. While frequencies of other lymphocyte lineages were similar to healthy controls, expression of markers related to differentiation, activation and exhaustion was distinct in HL.

The increase of EM T cells found in our cohort of patients with first diagnosis of early-stage unfavorable HL is in line with a recent study in both relapsed/refractory and newly diagnosed HL [24]. Similarly to our study, Cader et al. observed significantly fewer CD8^+^ naive T cells and CD56^bright^ NK cells in HL patients. In contrast, they also found lower expression of CD25 and CD62L as classical markers of T-cell activation, while we observed a higher expression of PD1 in our study. An upregulation of PD1 in various compartments of the TME and on circulating T cells is a common feature of HL and the disbalance between PD1 and other markers of T-cell activation most likely reflects T-cell exhaustion [24, 41, 42]. Strikingly, we found enhanced expression of several other markers of T-cell exhaustion in treatment-naive HL patients, which contrasts with other cancers [43]. The pattern of upregulated co-inhibitory molecules is overlapping with a recently described module of genes, which are co-regulated with PD1 in T-cell exhaustion [44]. Upregulated co-inhibitory molecules on peripheral lymphocytes included potentially druggable targets like LAG3, Tim3, or CTLA4, which are partially also highly expressed on residing lymphocytes in the TME of HL [45–47]. Based on our findings and preclinical data demonstrating synergistic effects with other treatments, evaluation of therapeutically targeting these molecules either as monotherapies or in combination appears highly promising in HL [48].

Only very few previous studies evaluated immune response to TAA in HL. The low tumor cell content of HL represents a major hurdle for next-generation sequencing technologies. Hence, detection of somatic mutations leading to nonsynonymous mutations was not feasible at the time our translational program was conducted. We therefore focused on five TAAs specifically expressed in the TME and shared between patients based on an extensive search in public databases and previous publications [29, 31, 32]. Previously, serological immune response to at least one out of 19 tested CTAs has been described in a Brazilian cohort of HL patients [31]. Dave et al. recently reported preliminary data of an ongoing study assessing antigen-specific T-cell responses to shared antigens in pediatric HL patients treated with anti-PD1 based 1st-line treatment [49]. While MAGE-A4 and PRAME were selected in both studies, Dave et al. additionally included SURVIVIN but not MAGE-C1, EBNA-1 and BMLF-1. Similar to our results, the frequency of patients responding to at least one of the analyzed TAAs did not differ significantly between pre- and post-treatment samples. The authors however described an increase of specific spots in enzyme-linked immunospot assays (Elispot), which we did not observe in our study. Assay differences and differences in clinical parameters such as patients’ age are likely explanations for the potentially higher overall rate of patients responding to at least one TAA (61% compared to 42% in our study) and observed differences in specific spots [49, 50]. The observed correlation of MAGE-A4 expression in pretreatment biopsies and detectable T-cell responses in FluoroSpot assays, suggests that tailored selection of TAAs might improve detectability of immune responses in HL. In post-hoc analysis of TAA expression in biopsies from HL patients, we identified CT45 and SURVIVIN as promising additional targets. Preliminary efficacy of TAA targeted therapy in HL has very recently been shown using adoptive T-cell therapy with combined targeting of multiple shared TAAs (PRAME, SSX2, MAGE-A4, SURVIVIN, and NY-ESO-1) by in-vitro expanded T cells [50].

In a previous publication, analysis of pre- and on-treatment re-biopsies taken within days after first dose of nivolumab revealed a striking very early clearance of malignant HRS cells and reshaping of the HL TME. This study suggested additional mechanisms (e.g., withdrawal of survival factors) of anti-PD1 treatment, as clonal expansion of T cells or enrichment of cytotoxic T cells in the TME could not be detected. However, the described rapid response is not contradictory to a fast reversion of an initially exhausted immune cell phenotype as described in this study. Studies interrogating sequential pre- and on-treatment paired blood and tissue samples are needed to determine the exact mechanisms behind early and long-term effects of anti-PD1 therapy in HL in the first-line or r/r setting. Especially when combined with T-cell receptor sequencing, specific T-cell expansion (e.g., MANAFEST T-cell assays [51]) and demonstration of cytotoxicity, these studies could generate substantial insights toward the underlying mechanisms in HL [19]. Factors determining susceptibility to anti-PD-1 treatment seem to be at least partly overlapping between HL and solid tumors as we found an association between expression of CXCL9, CCR5 and PD-L1 with early excellent response. Together with CD8A, CXCL13 and TRAF2, these genes were recently identified as predictors of response in a large, pooled analyses of patients treated with checkpoint inhibition in clinical trials.

One limitation of our study is that we had to restrict our analyses to a small number of TAAs due to the high number of lymphocytes needed in FluoroSpot assays. Future studies using disease-specific peptide pools including a more HL-specific set of TAAs may improve sensitivity of FluoroSpot assays. Due to the excellent response rate and ongoing PFS of patients treated within the NIVAHL, correlation of flow-cytometric data, ß2 M/HLA-II status and tumor-specific immune responses to PFS or other classical outcome parameters remains impossible. In the single patient with primary progressive disease during anti-PD1 monotherapy, baseline expression of PD1 on CD4^+^ T cells was lower (34.0% vs. 61.9% ± 23.1) and higher on CD8^+^ T cells (73.7% vs. 55.7% ± 14.8) while all other markers and the development during treatment was within the range of the total HL cohort. Of note, we did not observe any baseline or on-treatment response to any of the 5 TAA in this patient. Further studies ideally including single-cell based analyses are needed to elucidate the clinical relevance of immune responses to TAAs and also to neoantigens in HL. While of high clinical relevance and interest, analysis of correlates of immune-related adverse events was limited by sample size, event rate and sample availability in our study.

Taken together we found a highly exhausted phenotype of circulating lymphocytes, which was rapidly and durably reduced following anti-PD1 immune-checkpoint inhibition with nivolumab. The exceptionally high baseline expression of several co-inhibitory and -stimulatory molecules constitutes a possibility of peripheral immune monitoring and potentially promising therapeutic target for other emerging immune-checkpoint inhibitors beyond anti-PD1 inhibition in HL. Moreover, we frequently detected tumor-specific immune responses to TAA in HL, which can be exploited for optimized immune-monitoring in upcoming trials and as potential targets for immunotherapies.

## Supplementary information

Supplementary Figures / Tables Legends

Supplementary Figure 1

Supplementary Figure 2

Supplementary Figure 3

Supplementary Figure 4

Supplementary Figure 5

Supplementary Figure 6

Supplementary Figure 7

Supplementary Table 1

Supplementary Table 2
