# Supplementary material for: Reverted exhaustion phenotype of circulating lymphocytes as immune correlate of anti-PD1 first-line treatment in Hodgkin lymphoma
Source: Leukemia. 2021 Sep 28;36(3):760–71. doi: 10.1038/s41375-021-01421-z (PMC8885413; doi:10.1038/s41375-021-01421-z)
Supplement: Supplementary file 1 — Supplementary Figures / Tables Legends [file 41375_2021_1421_MOESM1_ESM.docx]

**Supplementary Figure Legends**

**Supplementary Figure 1. Consort Diagram**

**Supplementary Figure 2.** **Gating strategy for flow cytometry analysis.** Representative flow cytometry plots showing peripheral blood mononuclear cells of a healthy control sample. Dead cells were excluded (alive). Lymphocytes were selected by gating for size and granularity (Forward Scatter Area (FSC-A) versus Side Scatter Area (SSC-A), respectively). Living lymphocytes were further plotted by FSC Hight (FSC-H) versus FSC-A to gate single cells and exclude doublets. From the single cell gate (singles), CD45^+^ lymphocytes (CD45^+^) were gated based on CD45 expression. B cells were defined as CD45^+^ lymphocytes and the expression of CD19 (Bcells). T cells were defined by gating on CD45^+^ lymphocytes and the expression of CD3 (Tcells) whereas CD3^-^ cells expressing CD56 were considered as NK cells (NKcells). T cells were further divided into CD8 T cells (CD8Tcells) or CD4 T cells (CD4Tcells) based on their CD8 and CD4 expression, respectively.

**Supplementary Figure 3. Flow cytometric analyses revealed changes in T-cell maturation and reduced CD56^bright^ NK cells in PBMCs of treatment-naïve HL patients.** Peripheral blood mononuclear cells (PBMCs) of Hodgkin lymphoma (HL) patients before therapy (BT, n=72) and healthy donor PBMCs (HC, n=20) were analyzed by flow cytometry. **A,** Differences in the percentage of naïve (CCR7^+^CD45RA^+^) CD4 and CD8 T cells in PBMCs of HL patients before therapy and healthy control PBMCs. **B,** NK cells in percent of all CD3^-^ lymphocytes (detailed gating strategy in Supplementary Figure 2). CD56^bright^ and CD56^dim^ NK cells in percent of all CD3^-^ lymphocytes. Significant differences calculated by unpaired, two-tailed Mann-Whitney test are indicated by asterisks. * p≤ 0.05, ** p≤0.01, *** p≤0.001, **** p≤0.0001. Mean ± SD is indicated.

**Supplementary Figure 4. EBV^+^ and EBV^-^ Hodgkin lymphoma patients show similar percentages of lymphocyte subsets, T-cell activation and immune-inhibitory molecule expression.** Peripheral blood mononuclear cells (PBMCs) of Hodgkin lymphoma (HL) patients before therapy (BT, n=72) were analyzed by flow cytometry. The EBV status of 59 before therapy samples was available and patients were stratified to EBV^+^ (n=10) and EBV^-^ (n=49. Differences in lymphocyte subsets (**A**), T-cell activation (CD69^+^%Tcells, CD25^+^%Tcells and CD62L^+^%Tcells), T-cell differentiation (**B**)**,** CCR7^-^CD45RA^-^ effector memory (EM)), B-cell activation (CD86^+^%Bcells), B-cell differentiation (plasmablasts%Bcells) (**C**) and immune-regulatory molecule expression on T cells (**D,** PD1, LAG3, Tim3, OX40) between EBV^-^ and EBV^+^ patients are shown. Significant differences were calculated by unpaired, two-tailed Mann-Whitney test. Mean ± SD is indicated.

**Supplementary Figure 5. Patients from different treatment arms show similar cellular responses against tumor associated antigens.** Cellular immune responses against tumor associated antigens (TAA, BMLF-1, PRAME, MAGE-A4, MAGE-C1 and EBNA-1) were determined by FluoroSpot analysis of peripheral mononuclear cells (PBMCs) of Hodgkin lymphoma (HL) patients. Percentages of patients with (black) and without (grey) interferon gamma (IFN-y) responses against ≥ 1, ≥2 or ≥3 of the 5 tested TAAs are shown for patients from treatment arm A (**A**) and treatment arm B (**B**). **C,** Comparison of the number of patients with (black) and without (grey) IFN-y responses against ≥ 1, ≥2 or ≥3 of the 5 tested TAAs in patients from treatment arm A and B. **D,** Comparison of the number of patients with and without >90% MTV reduction and with the IFN-y response to at least one TAA for patients from treatment arm A, arm B and all patients (Arm A+B). Significant differences calculated with two-sided Fisher’s exact test are indicated.

**Supplementary Figure 6. RNA expression of tumor associated antigens in Hodgkin lymphoma patients (HL).** RNA expression of the indicated tumor associated antigens (TAAs) was assessed by NanoString based RNA expression analysis. Patient specific expression (dots) in the tumor and mean ± 95% confidence interval is indicated.

**Supplementary Figure 7. RNA expression pattern of patients with excellent treatment response.** RNA expression was determined by NanoString based RNA expression analysis. **A,** Transporter associated with antigen presentation 1 (TAP1) expression is indicated for patients with (n=63) or without (n=7) >90% MTV reduction and patients with (n=24) or without (n=35) IFN-y responses against at least one TAA (BMLF-1, PRAME, MAGE-A4, MAGE-C1 and EBNA-1). **B,** RNA expression of CCR5, CXCL9, PDL1, CD8A, CXCL13 and TRAF2 was analyzed in patients with or without >90% MTV reduction. Significant differences calculated by unpaired, two-tailed Mann-Whitney test are indicated by asterisks. * p≤ 0.05, ** p≤0.01. Mean ± 95% confidence interval is indicated.

**Supplementary Table Legends**

**Supplementary Table 1. Detailed information on antibodies used for flow cytometry and immunohistochemistry.**

**Supplementary Table 2. Percentages of lymphocyte subsets, T-cell maturation, T-cell activation an immune-regulatory molecule expression on T cells in beta-2 microglobulin (β2M) and human leukocyte antigen 2 (HLA-II) positive and negative Hodgkin lymphoma patients.** Percentages of subsets and marker expression of the indicated molecules in peripheral blood mononuclear cells (PBMCs) of Hodgkin lymphoma (HL) patients was determined by flow cytometry. Patients were stratified by their β2M (5 positive and 58 negative) or HLA-II (33 positive and 25 negative) status. Significant differences were calculated by unpaired, two-tailed Mann-Whitney test. Mean ± SD is indicated.
