# Supplementary material for: Reverted exhaustion phenotype of circulating lymphocytes as immune correlate of anti-PD1 first-line treatment in Hodgkin lymphoma
Source: Leukemia. 2021 Sep 28;36(3):760–71. doi: 10.1038/s41375-021-01421-z (PMC8885413; doi:10.1038/s41375-021-01421-z)

Supplementary Figure 1

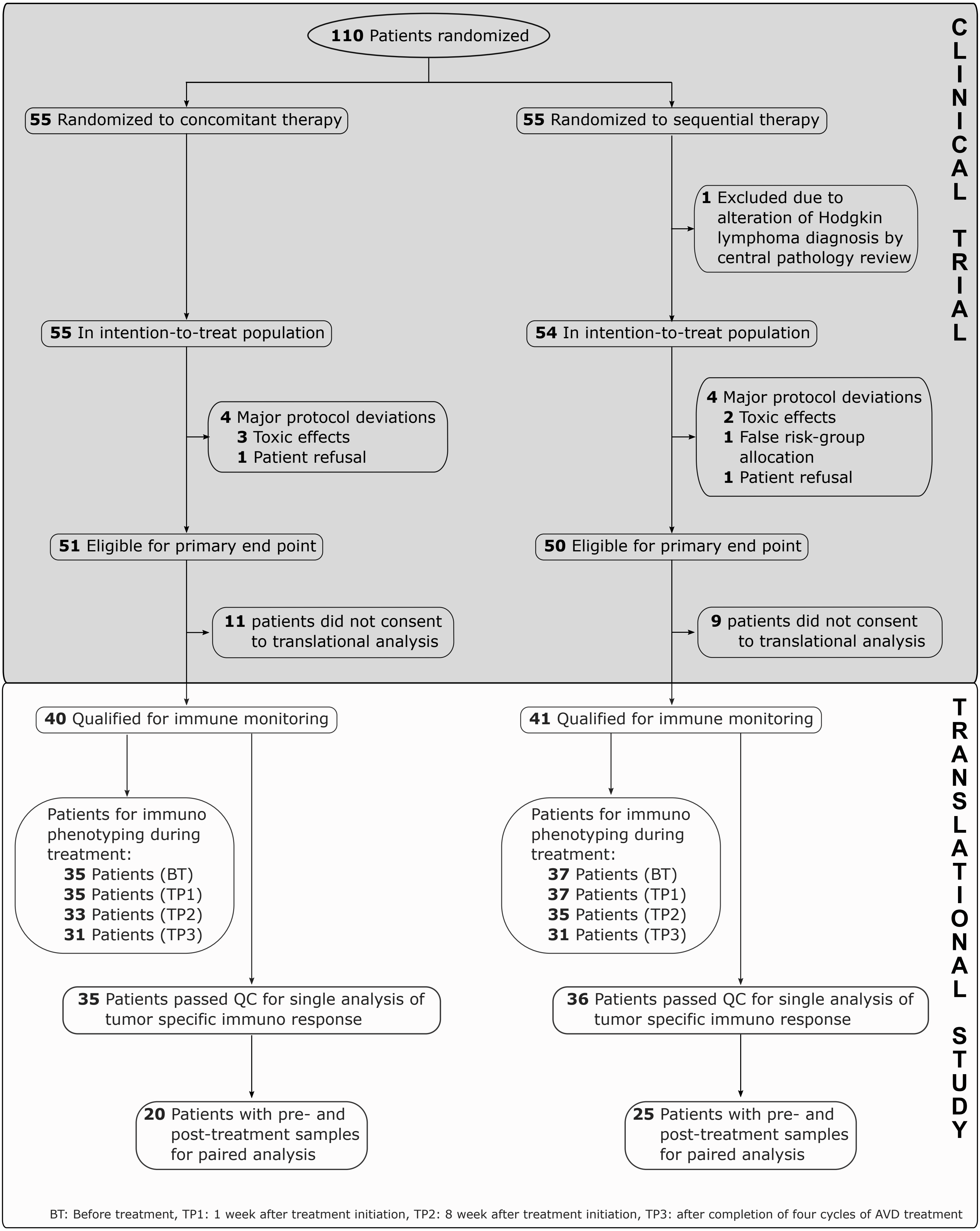

Supplement: Supplementary file 2 — Supplementary Figure 1 [file 41375_2021_1421_MOESM2_ESM.pdf]
