# Supplementary figures and images for: Reverted exhaustion phenotype of circulating lymphocytes as immune correlate of anti-PD1 first-line treatment in Hodgkin lymphoma
Source: Leukemia. 2021 Sep 28;36(3):760–71. doi: 10.1038/s41375-021-01421-z (PMC8885413; doi:10.1038/s41375-021-01421-z)

Supplementary Figure 2

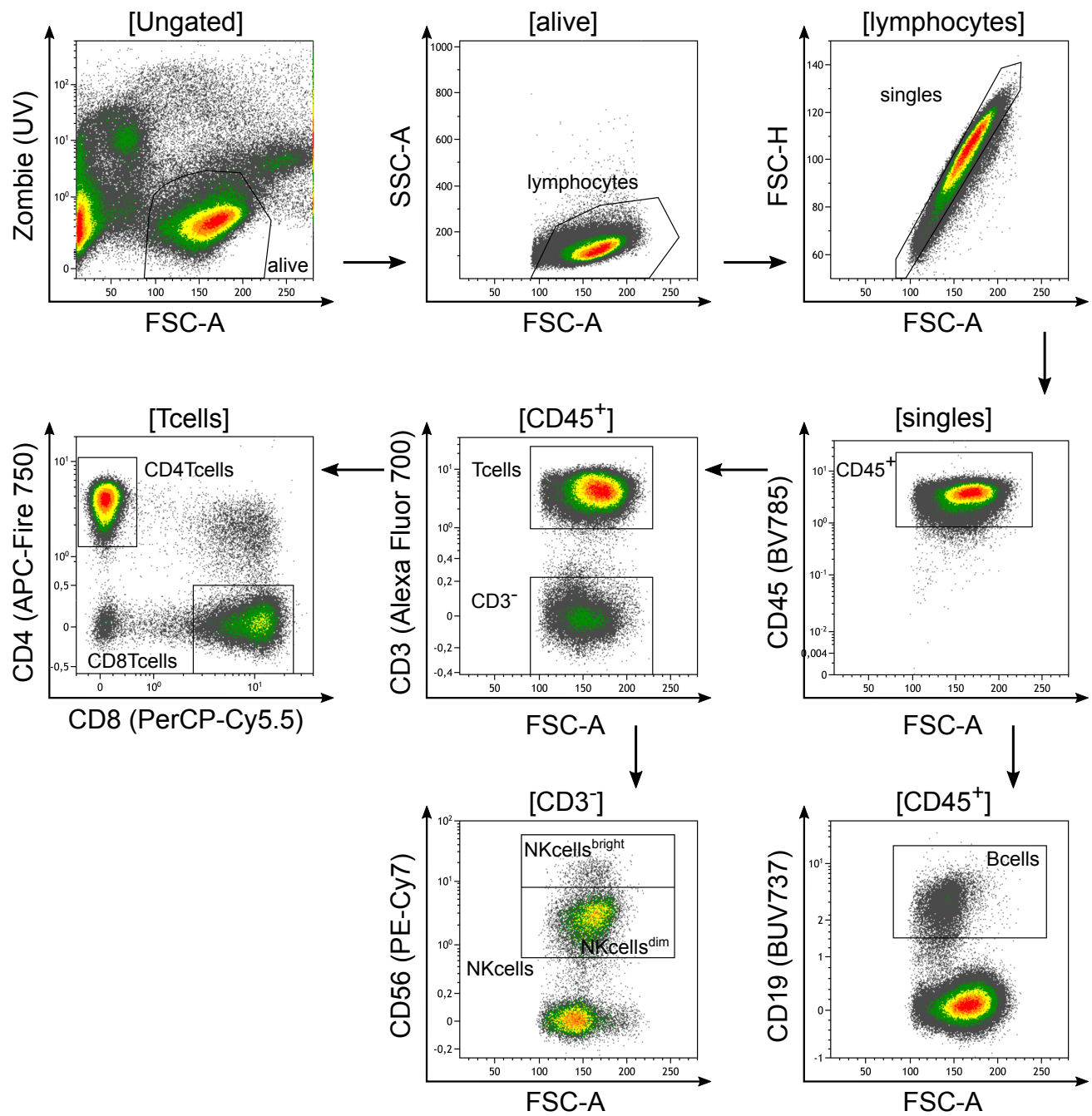

Supplement: Supplementary file 3 — Supplementary Figure 2 [file 41375_2021_1421_MOESM3_ESM.pdf]

Supplementary Figure 3

A

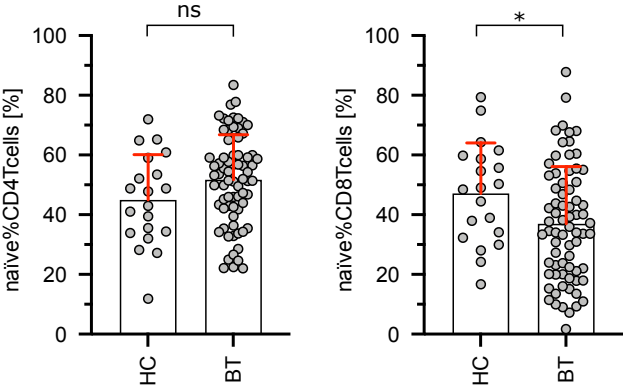

B

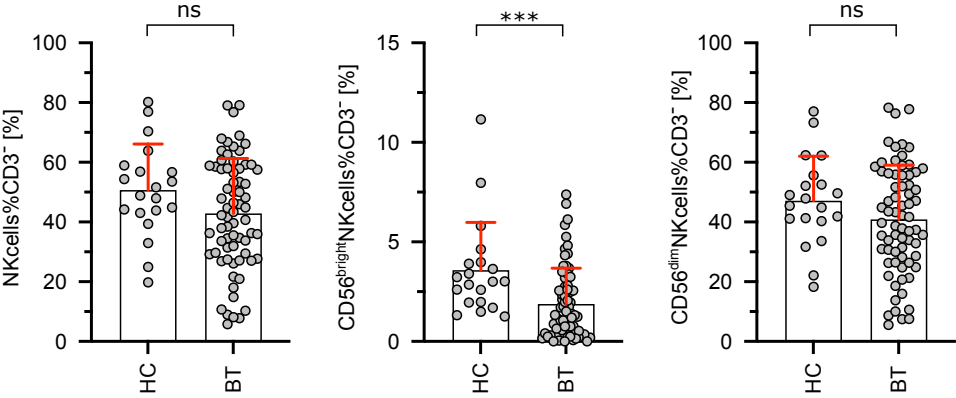

Supplement: Supplementary file 4 — Supplementary Figure 3 [file 41375_2021_1421_MOESM4_ESM.pdf]

Supplementary Figure 4

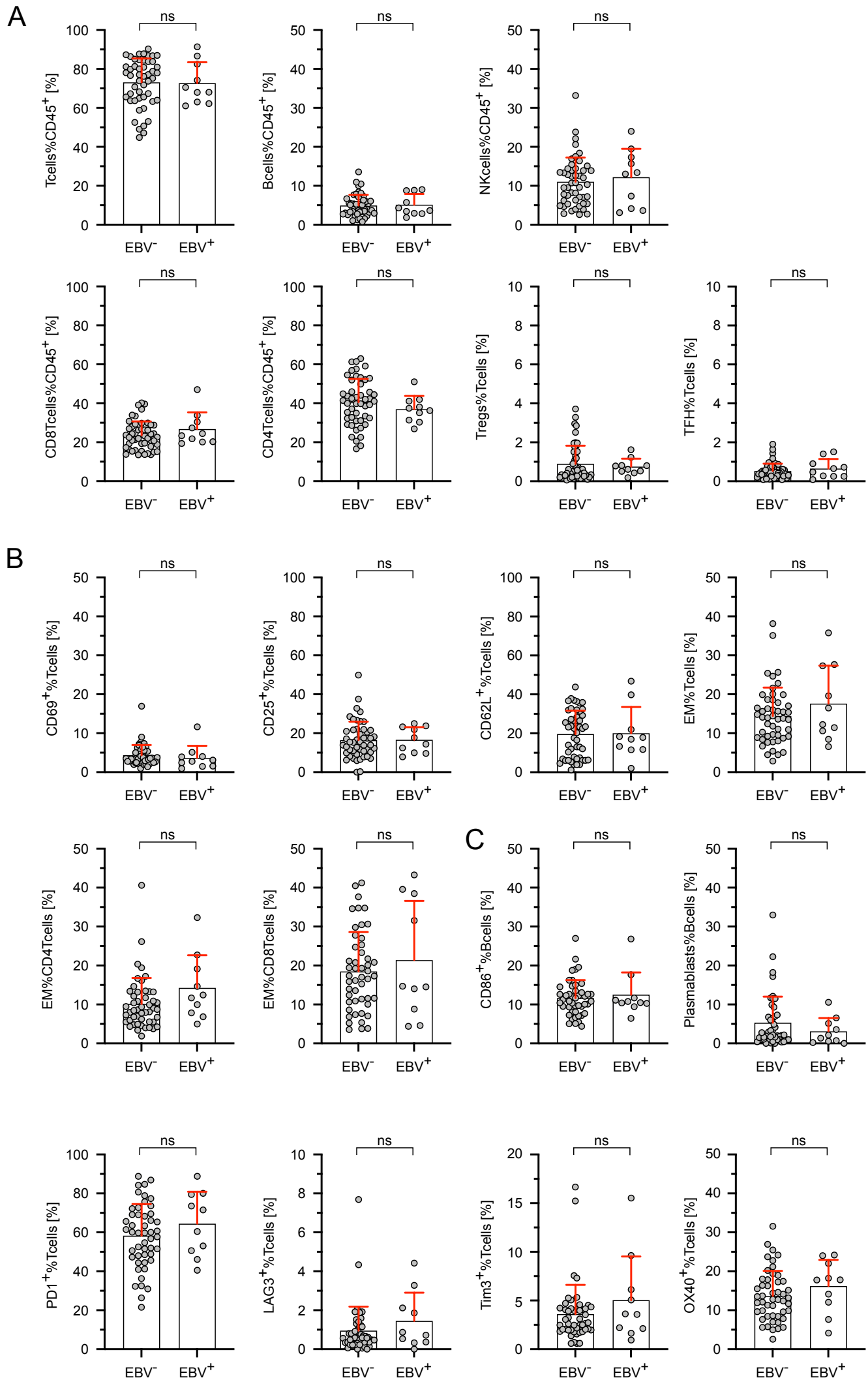

Supplement: Supplementary file 5 — Supplementary Figure 4 [file 41375_2021_1421_MOESM5_ESM.pdf]

Supplementary Figure 5

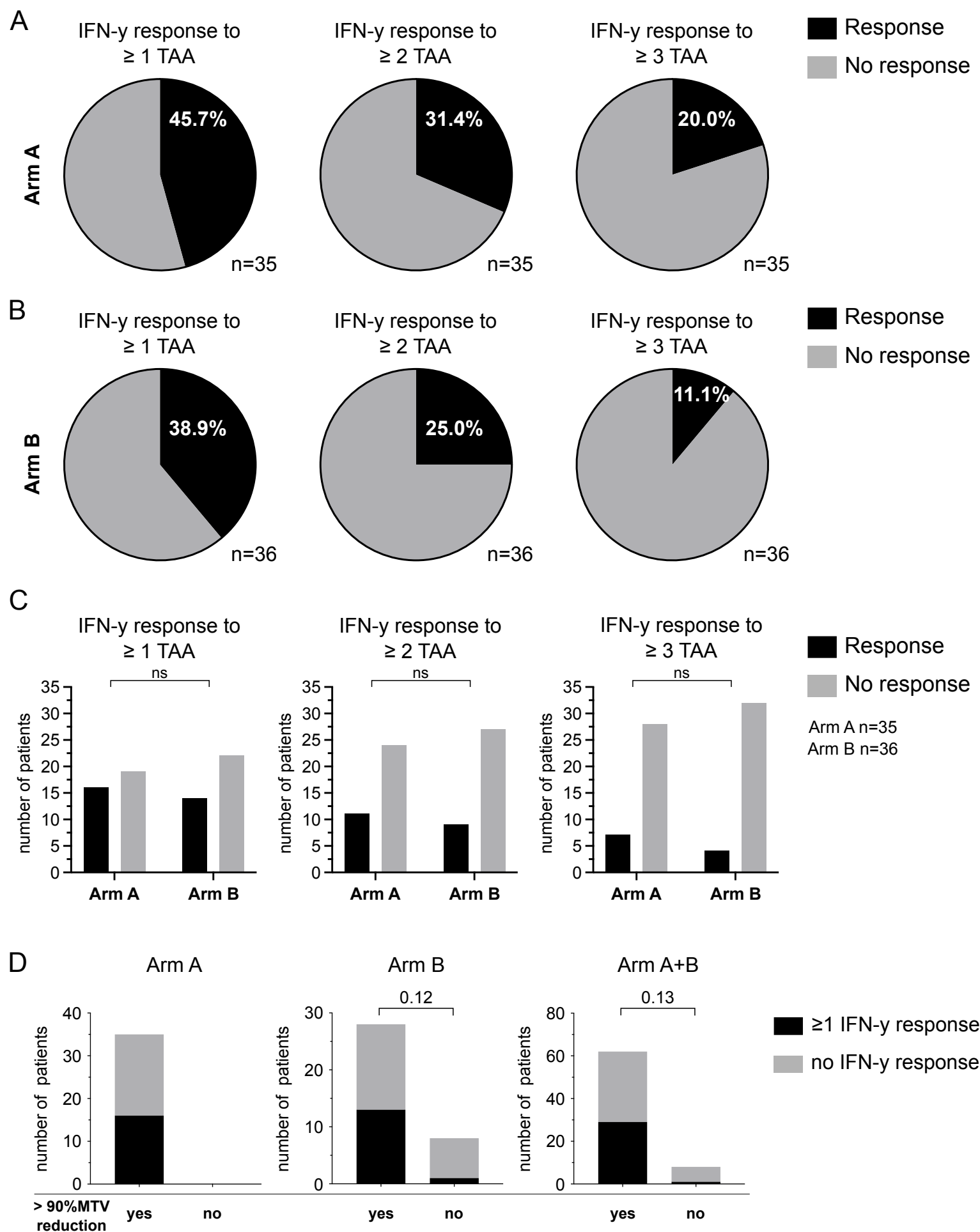

Supplement: Supplementary file 6 — Supplementary Figure 5 [file 41375_2021_1421_MOESM6_ESM.pdf]

Supplementary Figure 6

A

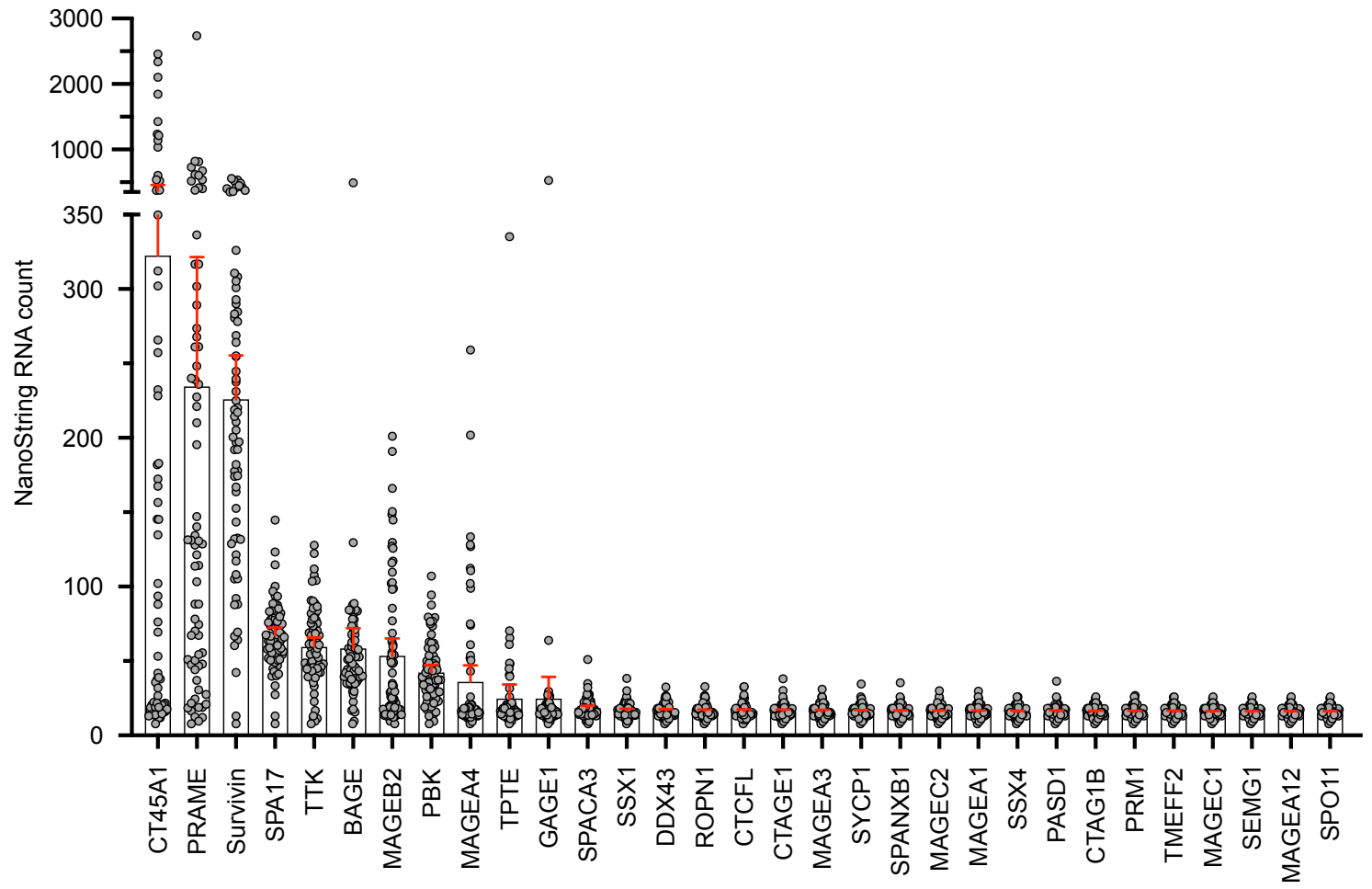

Supplement: Supplementary file 7 — Supplementary Figure 6 [file 41375_2021_1421_MOESM7_ESM.pdf]

Supplementary Figure 7

A

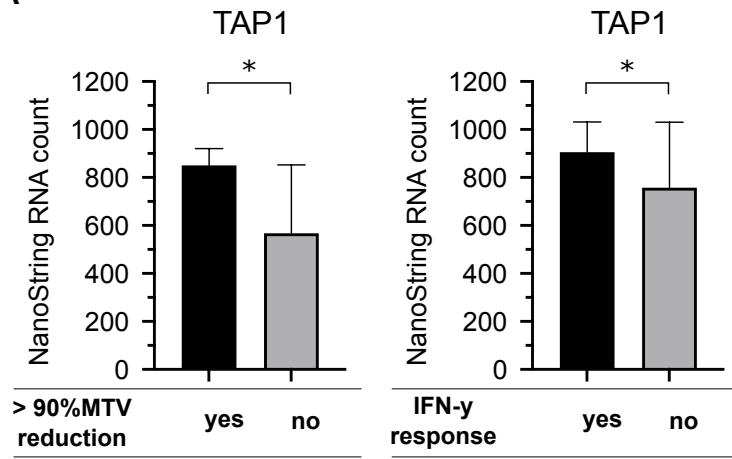

B

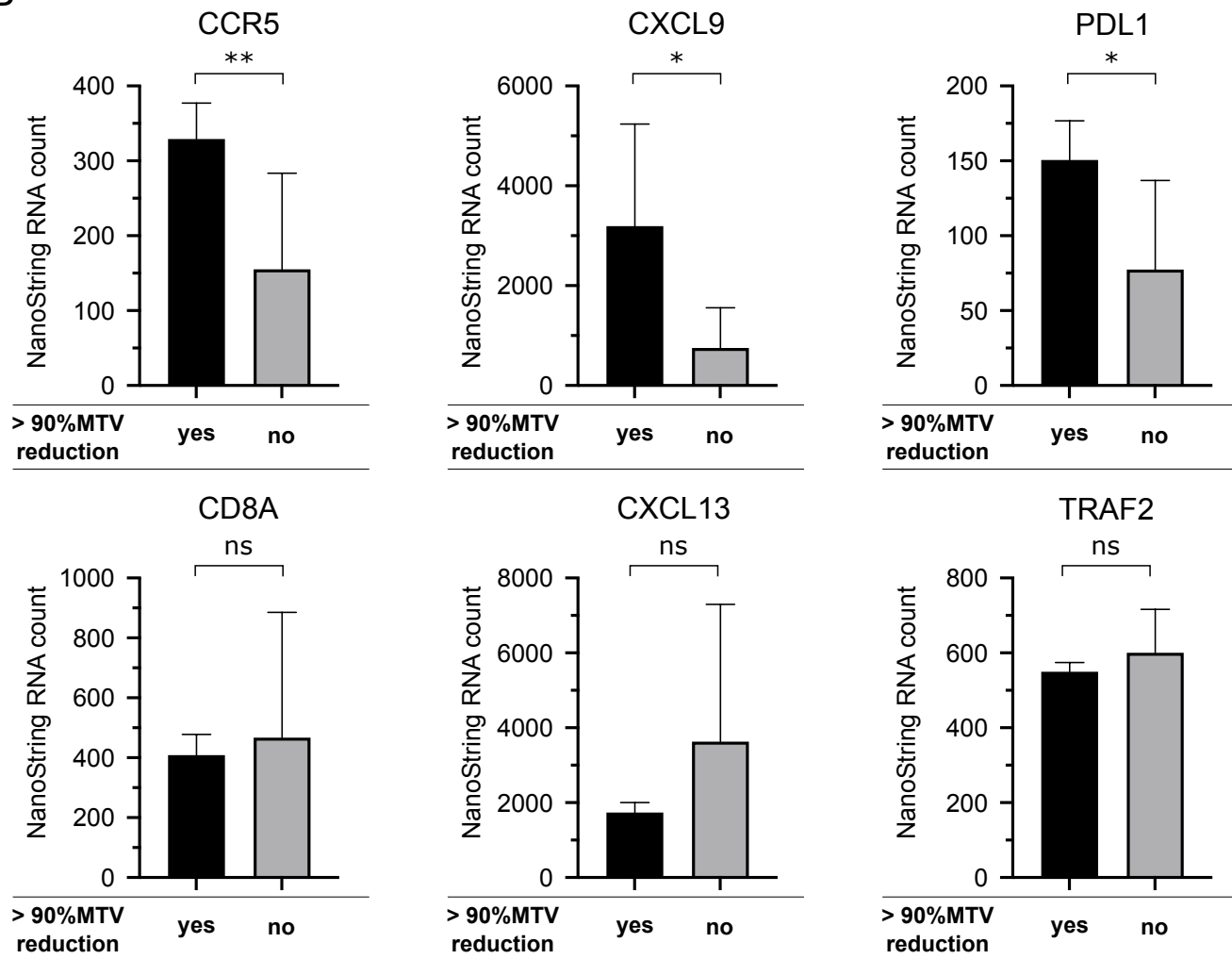

Supplement: Supplementary file 8 — Supplementary Figure 7 [file 41375_2021_1421_MOESM8_ESM.pdf]
