# Supplementary material for: Reverted exhaustion phenotype of circulating lymphocytes as immune correlate of anti-PD1 first-line treatment in Hodgkin lymphoma
Source: Leukemia. 2021 Sep 28;36(3):760–71. doi: 10.1038/s41375-021-01421-z (PMC8885413; doi:10.1038/s41375-021-01421-z)
Supplement: Supplementary file 9 — Supplementary Table 1 [file 41375_2021_1421_MOESM9_ESM.docx]

| **Flow Cytometry** | | | | |
| --- | --- | --- | --- | --- |
| **Fluorochrome** | **Target** | **Clone** | **Company** | **Catalogue** |
| Alexa Fluor 488 | CXCR5 | J252D4 | Biolegend | 356912 |
| FITC | CD8a | HIT8a | Biolegend | 300906 |
| FITC | CD20 | 2H7 | Biolegend | 302304 |
| FITC | CD62L | DREG-56 | Biolegend | 304804 |
| PerCP-Cy5.5 | CD8 | SK1 | Biolegend | 344710 |
| PerCP-Cy5.5 | CD38 | HIT2 | Biolegend | 303522 |
| PE | CD252 (OX40L) | 11C3.1 | Biolegend | 326308 |
| PE | CD96 (TACTILE) | NK92.39 | Biolegend | 338406 |
| PE | CD270 (HVEM) | 122 | Biolegend | 318806 |
| PE | CD258 (LIGHT) | T5-39 | Biolegend | 318706 |
| PE | CD48 | BJ40 | Biolegend | 336708 |
| PE | CD197 (CCR7) | G043H7 | Biolegend | 353204 |
| PE | CD152 (CTLA-4) | BNI3 | BD Bioscience | 555853 |
| PE | CD86 | IT2.2 | Biolegend | 305406 |
| PE-Dazzle | CD134 (OX40) | Ber-ACT35 | Biolegend | 350020 |
| PE-Dazzle | CD155 (PVR) | SKII.4 | Biolegend | 337616 |
| PE-Dazzle | CD154 (CD40L) | 24-31 | Biolegend | 310840 |
| PE-Dazzle | CD366 (Tim3) | F38-2E2 | Biolegend | 345034 |
| PE-Dazzle | CD244 (2B4) | C1.7 | Biolegend | 329522 |
| PE-Dazzle | CD25 | M-A251 | Biolegend | 356126 |
| PE-Dazzle | CD28 | CD28.2 | Biolegend | 302942 |
| PE-Dazzle | IgD | IA6-2 | Biolegend | 348240 |
| PE-Cy7 | CD56 | 5.1H11 | Biolegend | 362510 |
| PE-Cy7 | CD45RA | HI100 | Biolegend | 304126 |
| PE-Cy7 | CD73 | A2D | Biolegend | 344010 |
| PE-Cy7 | CD27 | O323 | Biolegend | 302838 |
| Alexa Fluor 647 | CD279 (PD1) | EH12.1 | BD Bioscience | 560838 |
| Alexa Fluor 647 | CD305 (LAIR1) | NKTA255 | Biolegend | 342802 |
| Alexa Fluor 647 | FoxP3 | 259D | Biolegend | 320214 |
| APC | CD226 (DNAM1) | 11A8 | Biolegend | 338312 |
| APC | NKG2A | REA110 | Miltenyi | 130-113-563 |
| APC | CD66ace | ASL-32 | Biolegend | 342308 |
| APC | CD44 | BJ18 | Biolegend | 338806 |
| APC | CD21 | Bu32 | Biolegend | 354906 |
| Alexa Fluor 700 | CD3 | SK7 | Biolegend | 344822 |
| APC-Fire 750 | CD4 | SK3 | Biolegend | 344638 |
| BV421 | TIGIT | A15153G | Biolegend | 372710 |
| BV421 | CD272 (BTLA) | MIH26 | Biolegend | 344512 |
| BV421 | CD223 (LAG3) | 11C3C65 | Biolegend | 369314 |
| BV421 | CD357 (GITR) | 108-17 | Biolegend | 371208 |
| BV421 | CD278 (ICOS) | C398.4A | Biolegend | 313524 |
| BV421 | CD39 | A1 | Biolegend | 328214 |
| BV421 | CD137 (41BB) | 4B4-1 | Biolegend | 309820 |
| BV421 | CD40 | 5C3 | Biolegend | 334332 |
| BV605 | CD154 (CD40L) | 24-31 | Biolegend | 310826 |
| BV605 | CD69 | FN50 | Biolegend | 310937 |
| BV605 | CD24 | ML5 | Biolegend | 311124 |
| BV785 | CD45 | HI30 | Biolegend | 304048 |
| Zombie UV | Fixable Viability Dye |  | Biolegend | 423108 |
| BUV737 | CD19 | SJ25C1 | BD Bioscience | 564303 |
| **Immunohistochemistry** | | | | |
| **Target** | **Clone** | **Dilution/ Device/ Retrieval** | **Company** | **Catalogue** |
| Beta-2M | polyclonal | 1:2000//manual staining//pH6 | DAKO | A007202-2 |
| HLA-DP/DQ/DR | CR3/43 | 1:100//manual staining//pH6 | DAKO | M077501-2 |
| LMP1 | CS.1-4 | 1:50//BondMax//* | DAKO | M0897 |
| * staining and scoring conducted by multiple GHSG reference pathologies as part of routine care | | | | |

Supplementary Table 1
